# Supplementary material for: AZGP1 in POMC neurons modulates energy homeostasis and metabolism through leptin-mediated STAT3 phosphorylation
Source: Nat Commun. 2024 Apr 20;15:3377. doi: 10.1038/s41467-024-47684-9 (PMC11032411; doi:10.1038/s41467-024-47684-9)
Supplement: Supplementary file 1 — Supplementary Information [file 41467_2024_47684_MOESM1_ESM.pdf]

AZGP1 in POMC neuron modulates energy homeostasis and metabolism through leptin-mediated STAT3 phosphorylation

Authors

Sheng Qiu[1,2#], Qinan Wu[3,#], Hao Wang[1,#], Dongfang Liu[1], Chen Chen[4], Zhiming Zhu[5], Hongting Zheng[6], Gangyi Yang[1*], Ling Li[1,2*], Mengliu Yang[1*]

Affiliations

[1]Department of Endocrinology, the Second Affiliated Hospital, Chongqing Medical University, Chongqing, 400010, China

[2]Department of Clinical Biochemistry and the Key Laboratory of Laboratory Medical Diagnostics in the Ministry of Education, Chongqing Medical University, Chongqing, 400016, China

[3]Department of Endocrinology, The affiliated Dazu hospital of Chongqing Medical university, Chongqing, 402360, China

[4]Endocrinology, SBMS, Faculty of Medicine, University of Queensland, Brisbane, 4072, Australia

[5]Department of Hypertension and Endocrinology, Daping Hospital, Third Military Medical University, Chongqing, 400042, China

[6]Department of Endocrinology, Xinqiao Hospital, Third Military Medical University, Chongqing, 400037, China

[#]These authors contributed equally to this work.

Correspondence: gangyiyang@hospital.cqmu.edu.cn (Gangyi Yang); liling@cqmu.edu.cn (Ling Li); mengliu.yang@cqmu.edu.cn (Mengliu Yang).

Supplementary Figures and Figure Legends

Supplementary Fig. 1

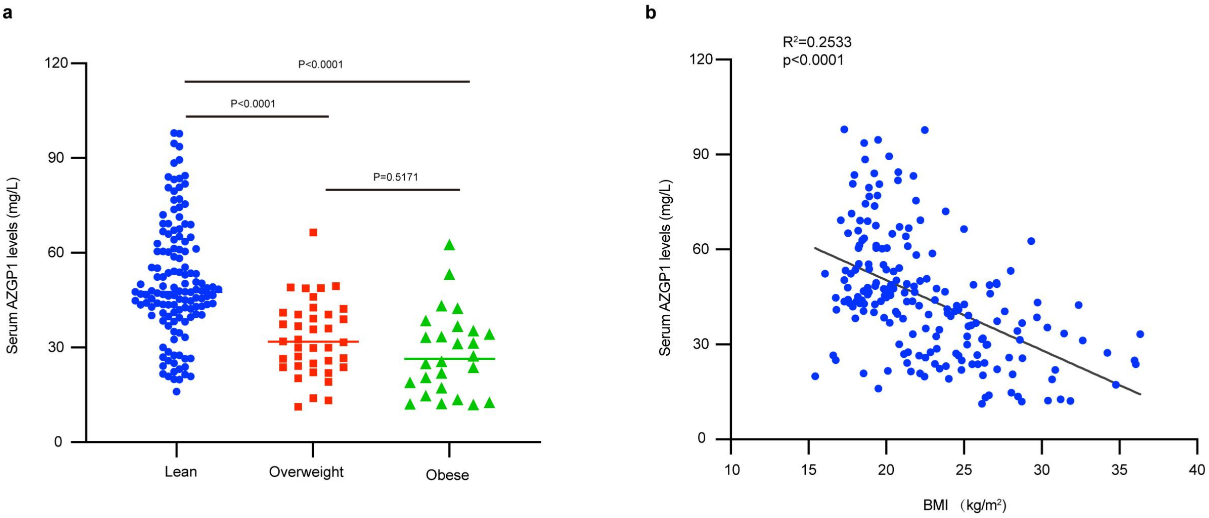

Supplementary Fig. 1 Serum AZGP1 levels are elevated in obese individuals and are associated with BMI. a Serum AZGP1 levels in lean individuals (BMI 18.5-23.9 kg/m^2, n = 135 independent individuals), overweight individuals (BMI 24-27.9 kg/m^2, n = 38 independent individuals) and obese individuals (BMI ≥ 28 kg/m^2, n = 26 independent individuals). **b** Correlation between AZGP1 levels and BMI. BMI, body mass index. The data are expressed as the mean ± SD. One-way ANOVA followed by Tukey's test **(a);** pearson correlation analysis **(b)**. Source data are provided as a Source Data file.

Supplementary Fig. 2

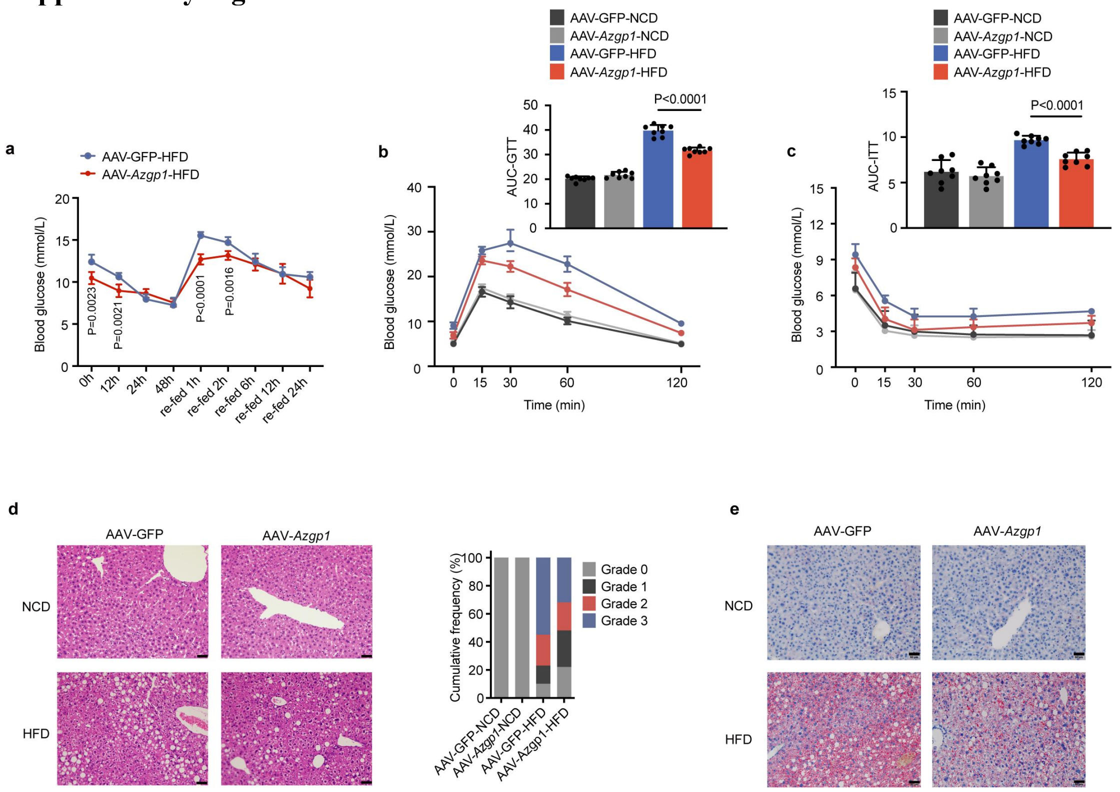

Supplementary Fig. 2 Effects of AZGP1 overexpression in the hypothalamus on glucose/lipid metabolism and insulin sensitivity. Eight-week-old male WT mice were injected with AAV9-*Azgp1*/GFP into the MBH and were fed a NCD or HFD for 12 weeks as described in the Methods. **a** Fasting and refeeding blood glucose levels in HFD-fed mice (n = 8 mice). **b, c** Blood glucose levels and the AUC of the GTT (**b**) and ITT (**c**) curves in NCD- and HFD-fed mice (n = 8 mice). **d** Representative H&E staining of the liver in NCD- and HFD-fed mice and quantitation of the grade of steatosis (n = 6 mice; scale bars: 50 μm). **e** Oil Red O staining of the liver in NCD- and HFD-fed mice (n = 6 mice; scale bars: 50 μm). GTT, glucose tolerance test; ITT, insulin tolerance test; AUC, area under the curve. The data are expressed as the mean ± SEM. Statistical significance was calculated using an unpaired two-tailed t-test **(a)** and two-way ANOVA followed by Bonferroni's post hoc tests **(b-c)**. Source data are provided as a Source Data file.

Supplementary Fig. 3

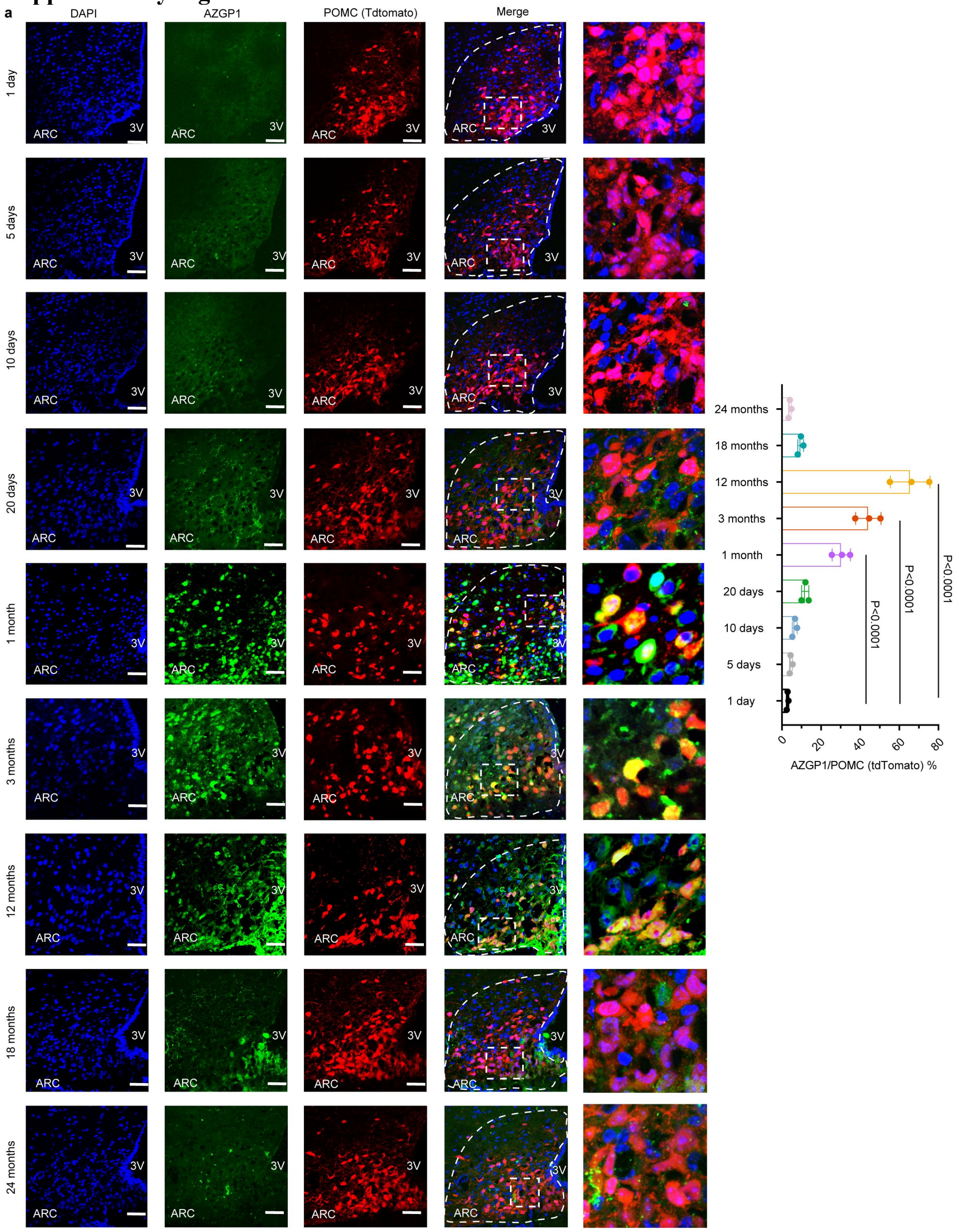

Supplementary Fig. 3 IF staining of AZGP1 in POMC neurons at different developmental stages in mice. (n = 3 mice); scale bars: 50 μm. ARC, arcuate nucleus; 3V, third cerebral ventricle. The data are expressed as the mean ± SEM. One-way ANOVA followed by Tukey's test **(a)**. Source data are provided as a Source Data file.

Supplementary Fig. 4

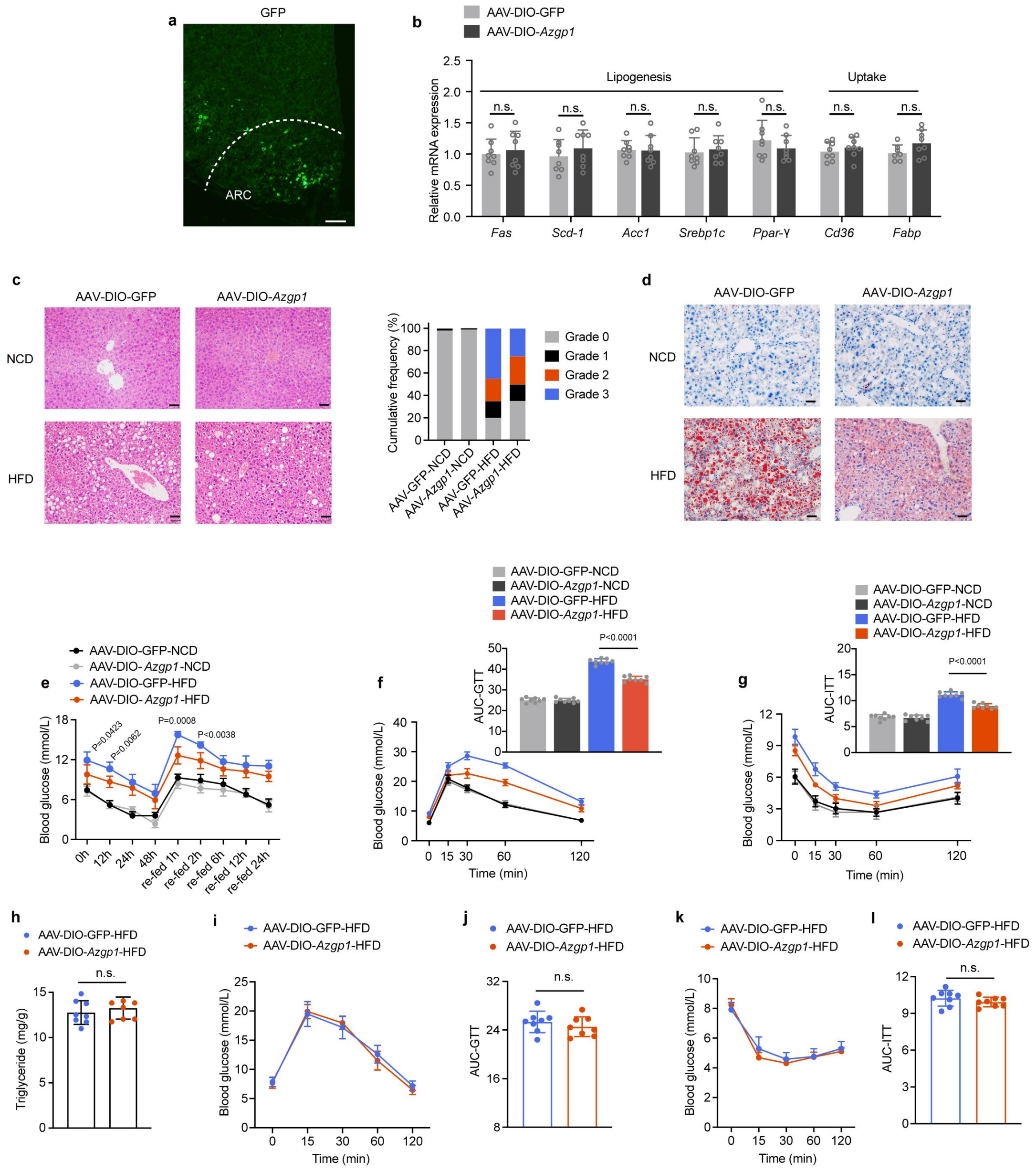

Supplementary Fig. 4 Effects of AZGP1 overexpression in POMC neurons on glucose/ lipid metabolism. Eight-week-old male POMC-Cre mice received bilateral injections of AAV9-DIO-*Azgp1*/GFP into the MBH and were fed a NCD or HFD for 12 weeks as described in the Methods. **a** Representative IF image showing GFP expression in the mouse hypothalamus (n = 3 mice). **b** Lipid metabolism-related gene expression in eWAT in HFD-fed mice (n = 8 mice). **c** Representative H&E staining of liver sections and quantitation of the grade of steatosis (n = 5 mice; scale bars: 50 μm). **d** Oil Red O staining of representative liver sections (n = 5 mice; scale bars: 50 μm). **e** Fasting and refeeding blood glucose levels (n = 9

mice). **f, g** Blood glucose levels and the AUC of the GTT (**f**) and the ITT curves (n = 8 mice). **h-l** Eight-week-old male POMC-Cre mice were given injections of AAV9-DIO-*Azgp1*/GFP into the bilateral MBH and were fed a HFD for 4 weeks. (**h**) TG levels in the liver (n = 8 mice). (**i-l**) Blood glucose levels and the AUC of the GTT and ITT curves (n = 8 mice). ARC, arcuate nucleus; VMH, ventromedial nucleus; 3V, third cerebral ventricle; GTT, glucose tolerance test; ITT, insulin tolerance test; AUC, area under the curve. The data are expressed as the mean ± SEM. Two-tailed Student's t tests were used in **(b), (h–l)**, and two-way ANOVA with Bonferroni post-hoc tests were used in **(e-g)**. Source data are provided as a Source Data file. (n.s. not significant.).

Supplementary Fig. 5

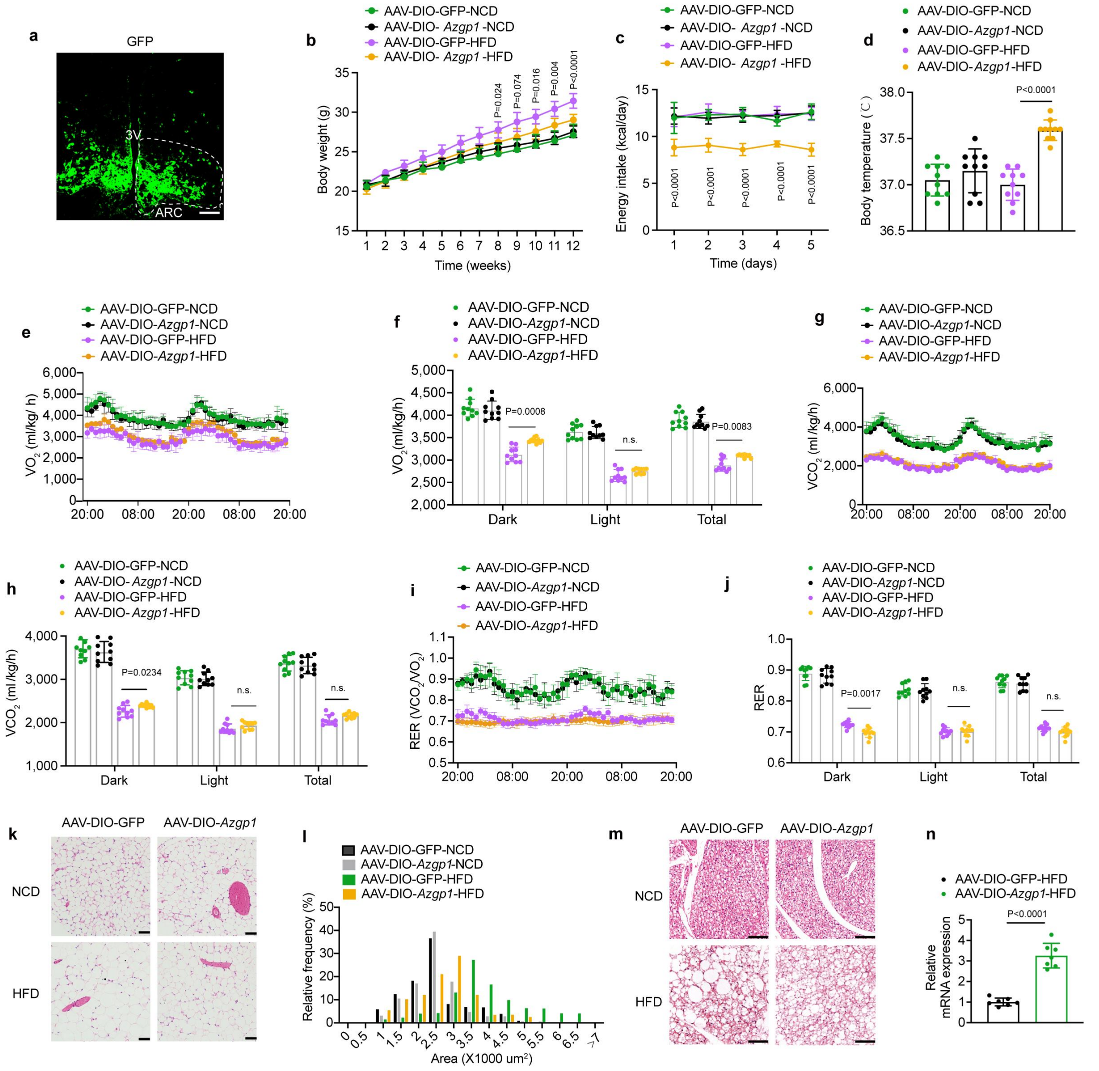

Supplementary Fig. 5 Specific overexpression of AZGP1 in POMC neurons increases energy expenditure and ameliorates metabolic disorders in HFD-fed female mice. Eight-week-old female POMC-Cre mice were injected with AAV9-DIO-*Azgp1*/GFP into the bilateral MBH and were fed a NCD or HFD for 12 weeks as described in the Methods. **a** Representative IF image showing GFP expression in the ARC. **b** Body weight curve (n = 8 mice). **c** Energy intake (n = 10 mice). **d** Rectal temperature (n = 10 mice). **e, f** Forty-eight hours oxygen consumption (n = 10 mice). **g, h** Forty-eight hours carbon dioxide consumption (n = 10 mice). **i, j** Respiratory exchange ratio (RER, VCO_2/VO_2) (n = 10 mice). **k** Representative H&E staining image of eWAT (n = 5 mice; scale bar: 50 μm). **l** Cross-sectional area of eWAT quantified by ImageJ (n = 5 mice). **m** Representative H&E staining image of

BAT (n = 5 mice; scale bar: 100 µm). **n** The mRNA expression of *Ucp1* (n = 7 mice). ARC, arcuate nucleus; 3V, third cerebral ventricle. The data are expressed as the mean ± SEM. Two-way ANOVA followed by Holm-Šídák multiple comparisons test **(b, c; f, h, j);** One-way ANOVA followed by Tukey's test **(d)**； Two-tailed Student's t test **(n)**. Source data are provided as a Source Data file. (n.s. not significant.).

Supplementary Fig. 6

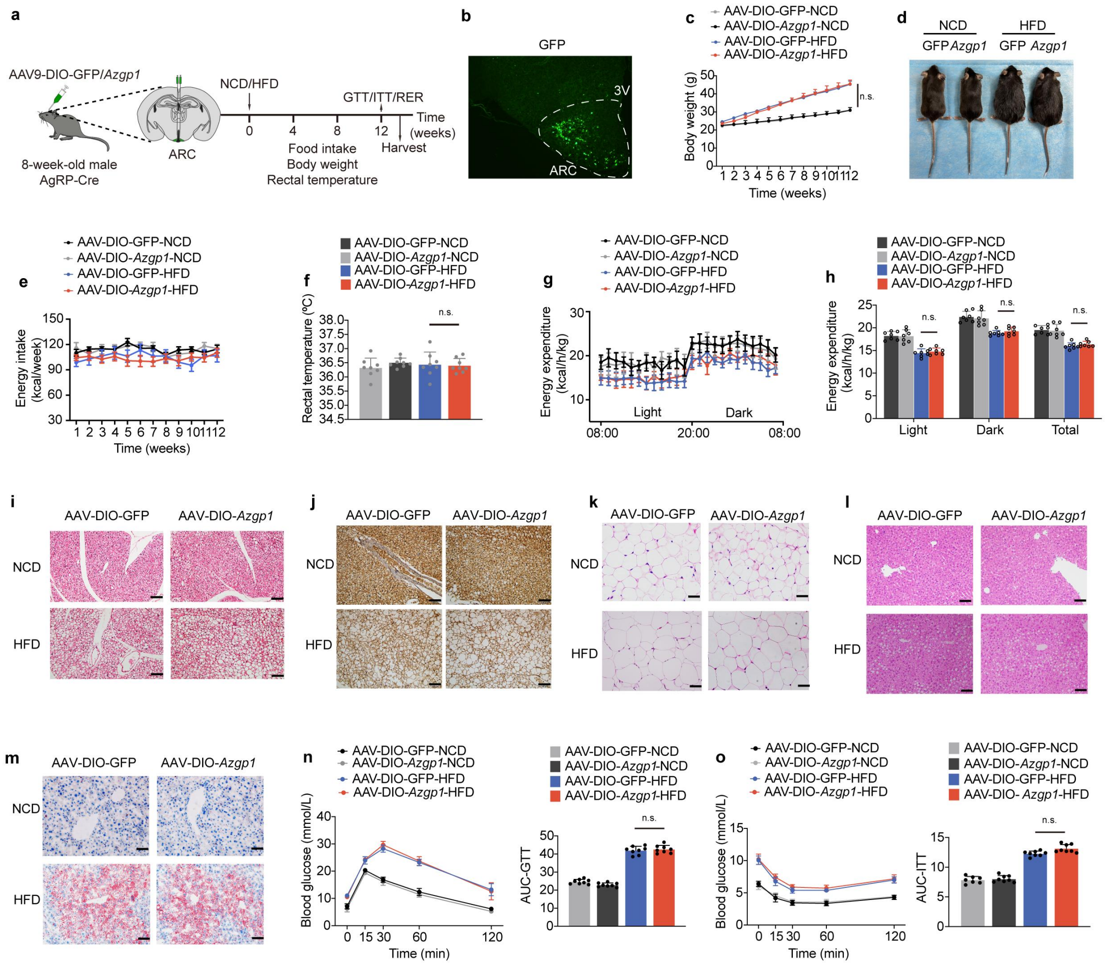

Supplementary Fig. 6 Overexpression of AZGP1 in AgRP neurons did not affect energy balance. a Schematic diagram of the experimental procedure. **b** IF staining image showing GFP expression in the ARC. **c** Body weight curve (n = 8 mice). **d** Representative photograph of mice. **e** Energy intake (n = 7 mice). **f** Rectal temperature (n = 8 mice). **g, h** Energy expenditure (n = 7 mice). **i** H&E staining of BAT (n = 5 mice; scale bars: 100 μm). **j** UCP1 immunostaining of BAT (n = 5 mice; scale bars: 100 μm). **k** H&E staining of eWAT (n = 5 mice; scale bars: 50 μm). **l** H&E staining of the liver (n = 5 mice; scale bars: 50 μm). **m** Oil Red O staining of the liver (n = 5 mice; scale bars: 50 μm). **n, o** Blood glucose levels and the AUC of the GTT (**n**) and ITT (**o**) curves (n = 8 mice). ARC, arcuate nucleus; 3V, third cerebral ventricle; GTT, glucose tolerance test; ITT, insulin tolerance test; AUC, area under the curve. The data are expressed as the mean ± SEM; one-way ANOVA followed by Tukey's

test **(f)**, two-way ANOVA followed by Bonferroni's post hoc tests **(c, e; h; n; o)**. Source data are provided as a Source Data file. (n.s. not significant.).

Supplementary Fig. 7

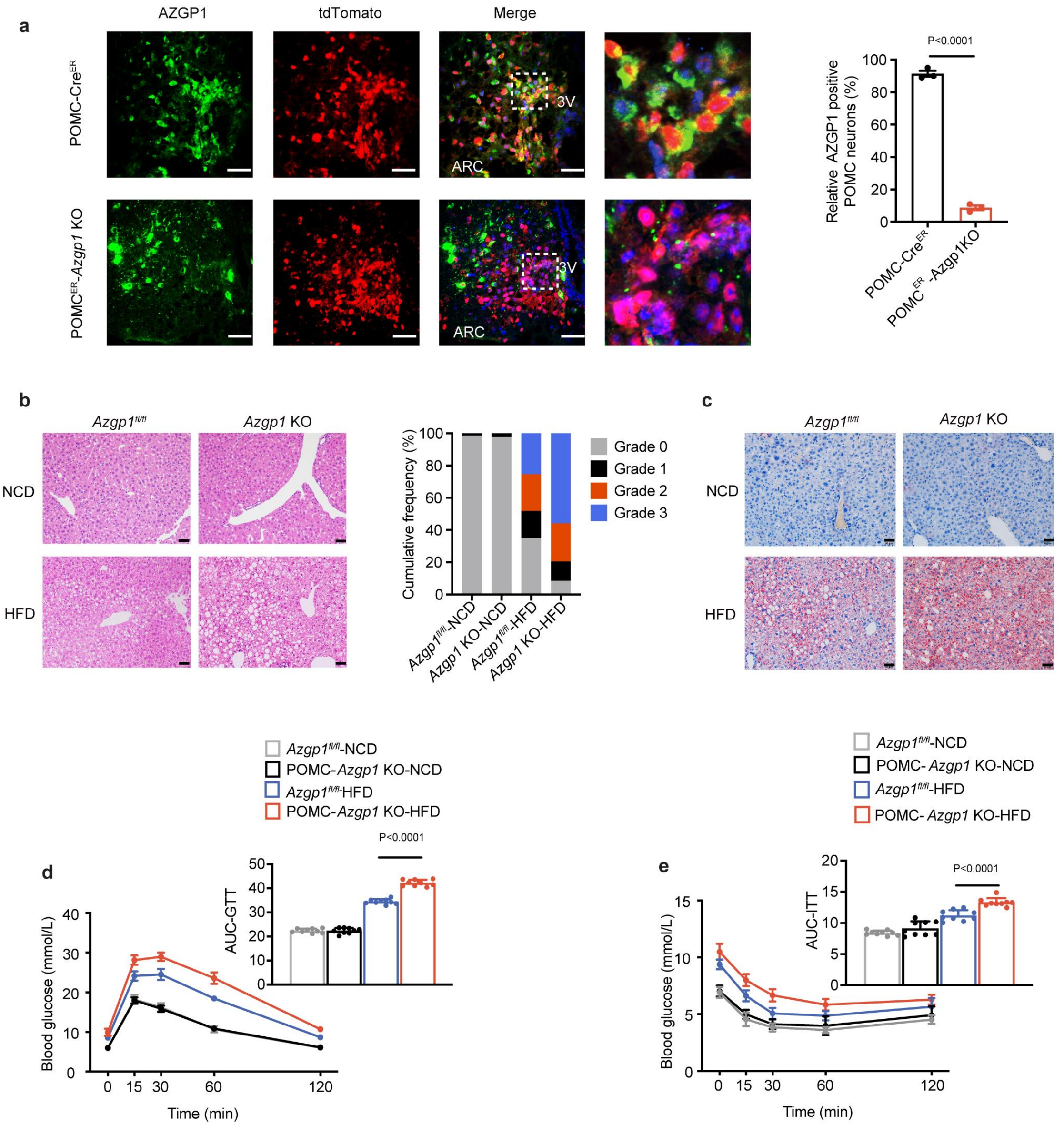

Supplementary Fig. 7 Effects of *Azgp1* ablation in POMC neurons on lipid metabolism. **a** IF staining of AZGP1 in the ARC POMC neurons (tdTomato) of POMC-*Azgp1* KO or POMC-Cre^{ER} mice (n = 3 mice; scale bars: 50 μm). **b, c** POMC-*Azgp1* KO and *Azgp1*$^{fl/fl}$ mice were fed a NCD or HFD for 12 weeks. H&E staining of liver sections and quantitation of the grade of steatosis (**b**) (n = 5 mice; scale bars: 50 μm). **c** Oil Red O staining of liver sections (n = 5 mice; scale bars: 50 μm). **d, e** Blood glucose levels and the AUC of the GTT (**d**) and the ITT (**e**) curves (n = 9 mice). ARC, arcuate nucleus; 3V, third cerebral ventricle; GTT, glucose tolerance test; ITT, insulin tolerance test; AUC, area under the curve. The data are expressed as the mean ± SEM. Two-tailed Student's t tests were used in **(a)** and two-way ANOVA with

Bonferroni post-hoc tests were used in **(d), (e)**. Source data are provided as a Source Data file.

Supplementary Fig. 8

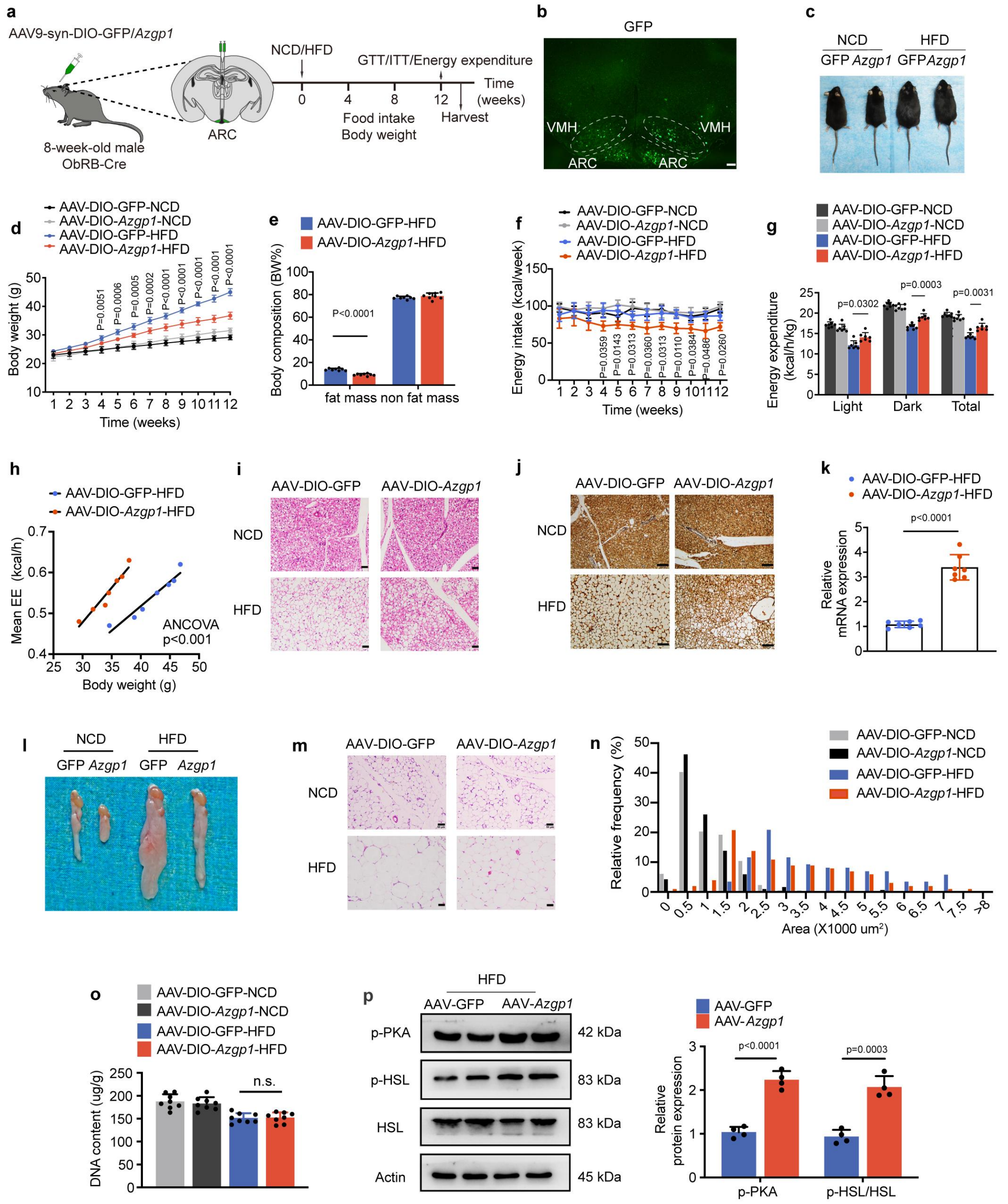

Supplementary Fig. 8 Overexpression of AZGP1 in ObRb neurons promotes energy expenditure in HFD-fed mice. a Schematic diagram of the experimental procedure. **b** IF staining of GFP expression in the ARC and VMH (n = 3 mice; scale bars: 200 μm). **c** A representative photograph of mice. **d** Body weight curve (n = 7 mice). **e** Body composition (n = 8 mice). **f** Energy intake (n = 5 mice). **g** Energy expenditure (n = 7 mice) . **h** ANCOVA of energy expenditure versus body weight in HFD-fed mice (n = 7 mice). **i** Representative H&E staining images of BAT (n = 5 mice; scale bars: 50 μm). **j** UCP1 immunostaining in BAT (n =

5 mice; scale bars, 100 μm). **k** *Ucp1* mRNA expression in BAT (n = 7 mice). **l** Representative image of eWAT depots. **m** Representative H&E staining image of eWAT (n = 5 mice; scale bars: 50 μm). **n** Cross-sectional area of eWAT quantified by ImageJ (n = 5 mice; scale bars: 50 μm). **o** DNA content in eWAT (n = 8 mice). **p** Western blot analysis of p-PKA and p-HSL/HSL expression in eWAT of HFD-fed mice and densitometric quantification (n = 4 mice). ARC, arcuate nucleus; VMH, ventromedial nucleus. The data are expressed as the mean ± SEM. Two-tailed Student's t tests were used in **(e, k, o, p)**, one-way ANCOVA using body weight as covariate **(h)** and two-way ANOVA with Bonferroni post-hoc tests were used in **(d, f, g,)**. Source data are provided as a Source Data file. (n.s. not significant.).

Supplementary Fig. 9

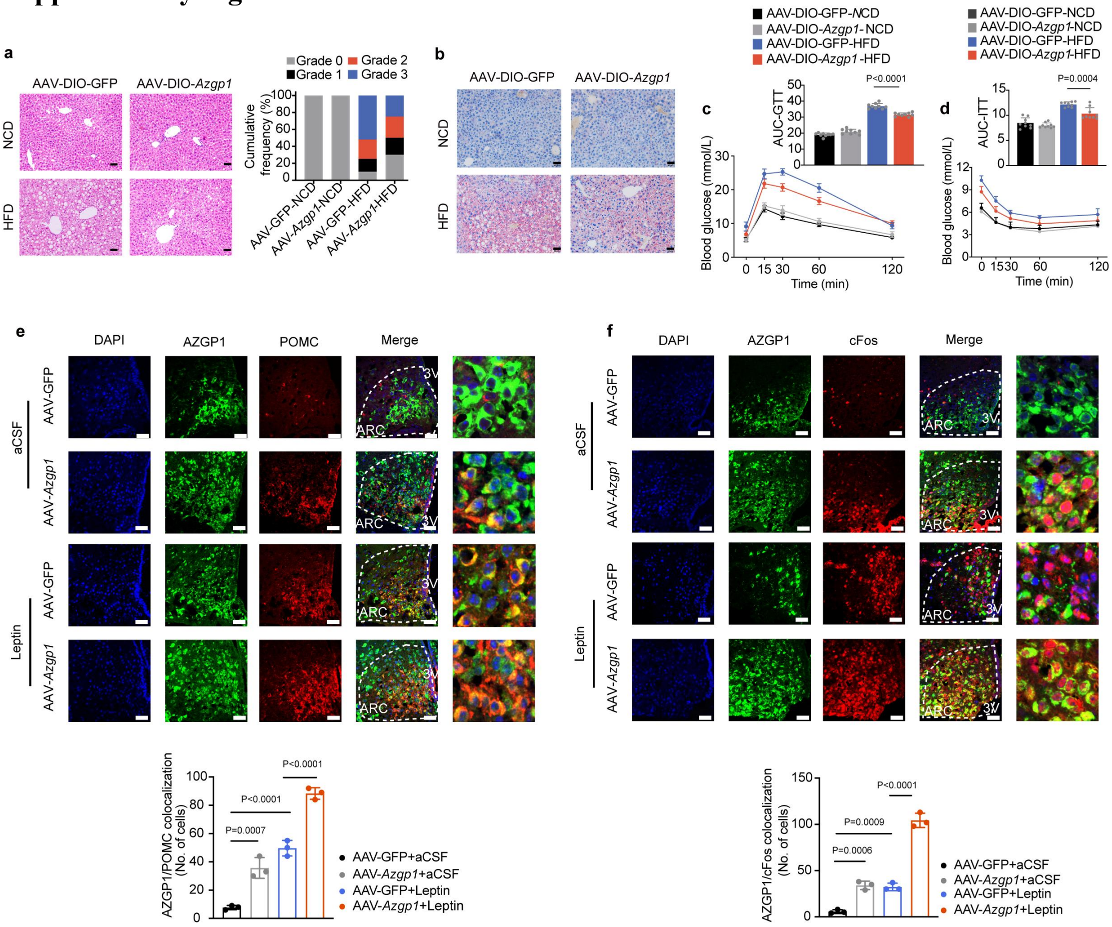

Supplementary Fig. 9 Effects of AZGP1 overexpression in ObRb neurons on glucose/ lipid metabolism and the excitability of neurons. Eight-week-old male ObRb-Cre mice were injected with AAV9-DIO-*Azgp1*/GFP into the MBH and fed a NCD or HFD for 12 weeks. **a** Representative H&E staining of liver sections and quantitation of the grade of steatosis (n = 5 mice; scale bars: 50 μm). **b** Oil Red O staining of the liver sections (n = 5 mice; scale bars: 50 μm). **c, d** Blood glucose levels and the AUC of the GTT (**c**) and ITT (**d**) curves (n = 9 mice). **e, f** IF staining for Azgp1 and POMC expression (**e**), and cFos (**f**) in the ARC of HFD-fed mice with or without MBH leptin (n = 3 mice; Scale bars: 50μm). ARC, arcuate nucleus; 3V, third cerebral ventricle; GTT, glucose tolerance test; ITT, insulin tolerance test; AUC, area under the curve. The data are expressed as the mean ± SEM. Two-way ANOVA with Bonferroni post-hoc tests were used in **(c–f)**. Source data are provided as a Source Data file.

Supplementary Fig. 10

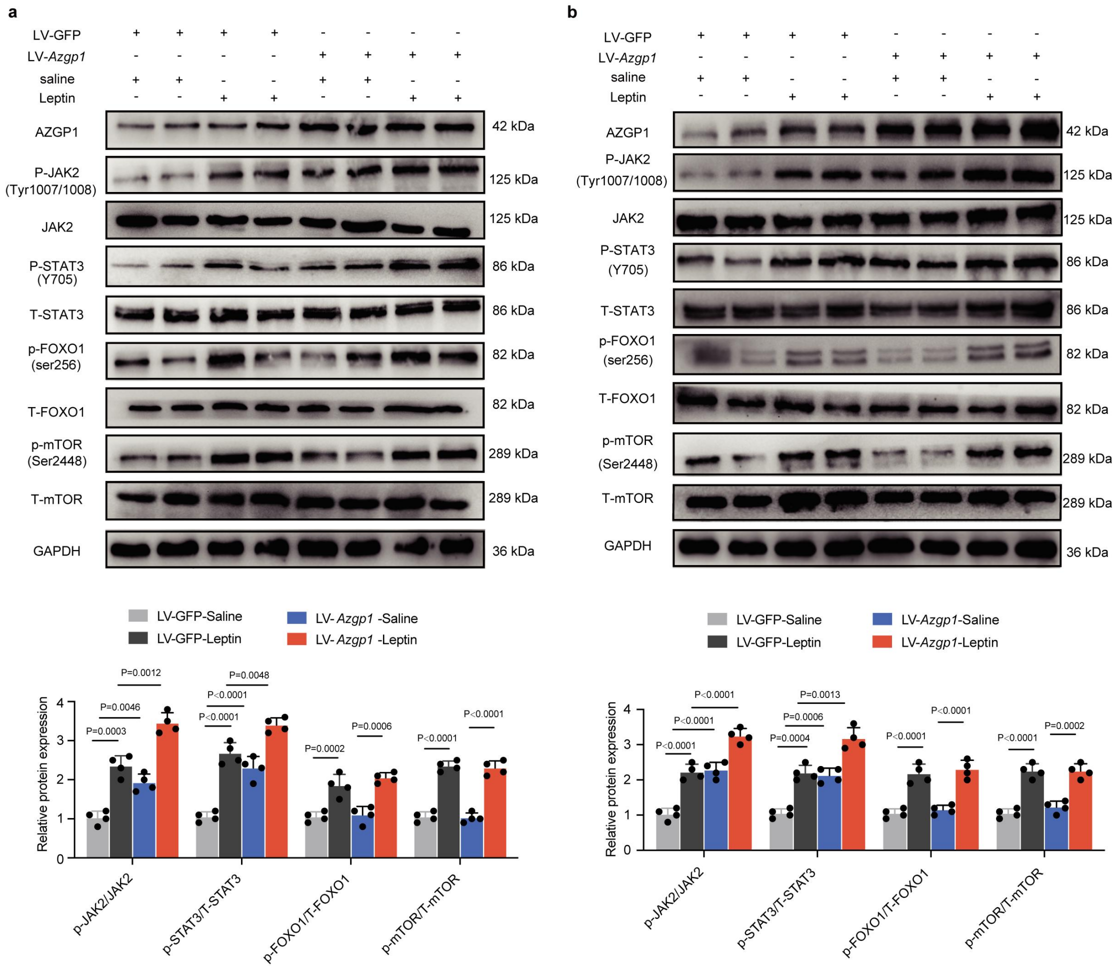

Supplementary Fig. 10 Overexpression of AZGP1 increases leptin-mediated STAT3 phosphorylation *in vitro.* GT1-7 and N2A cells were transfected with LV-*Azgp1*/GFP and treated with or without leptin as described in the Methods section. **a, b** Western blot analysis of the expression of metabolism-related signaling pathway components in GT1-7 (**a**) and N2A (**b**) cells (n = 4 independent cell experiments). The data are expressed as the mean ± SEM. Two-way ANOVA with Bonferroni post-hoc tests were used in **(a, b)**. Source data are provided as a Source Data file.

Supplementary Fig. 11

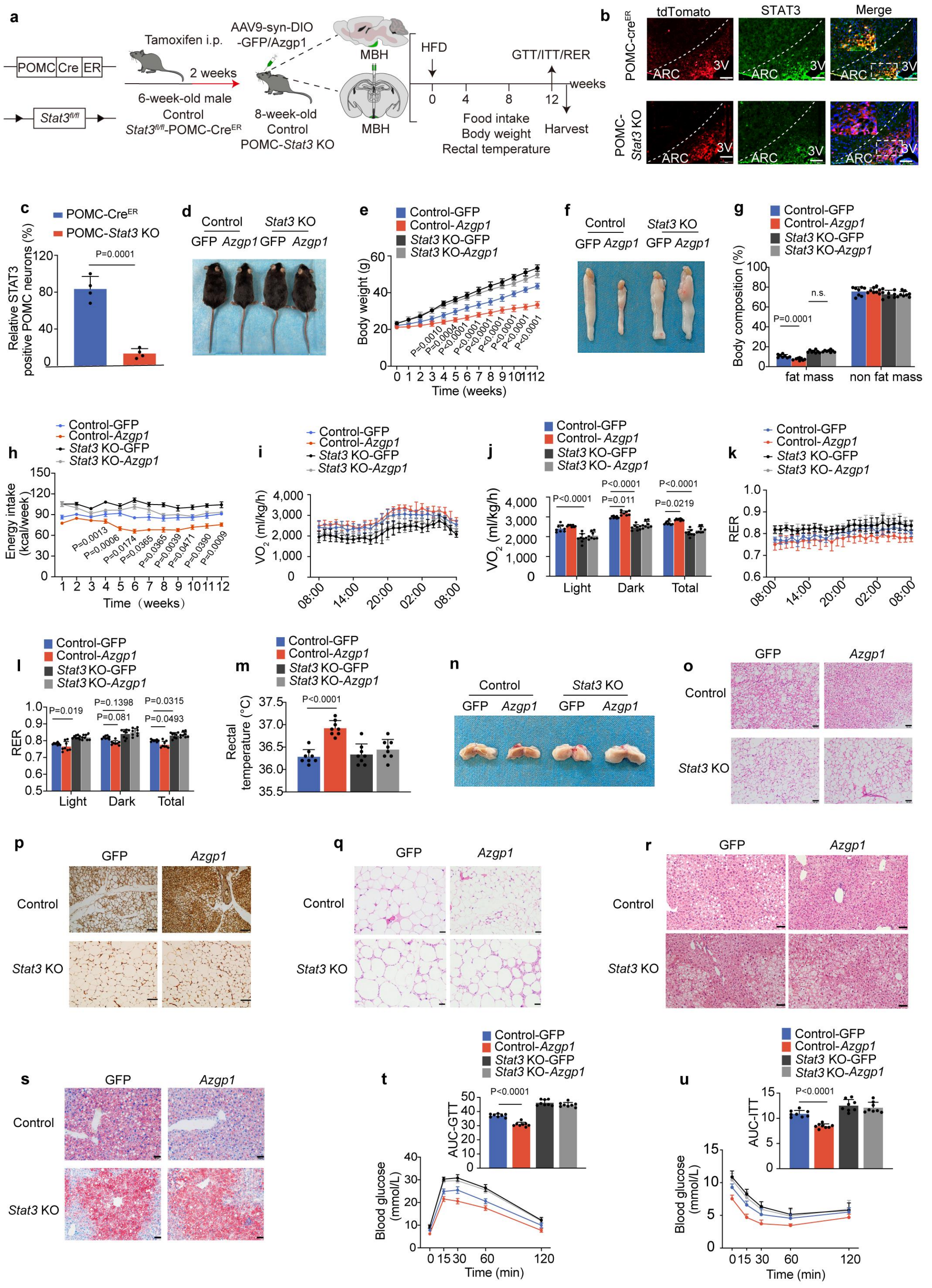

Supplementary Fig. 11 Inducible deletion of *Stat3* in POMC neurons abolishes the effect of central AZGP1 on energy metabolism. a Schematic diagram of the experimental procedure. **b** IF images showing STAT3 in POMC neurons (tdTomato) in the ARC (n = 4

mice; scale bars: 100 μm). **c** Densitometric quantification of STAT3 expression in **(b)**. **d** Representative photograph of mice. **e** Body weight curve (n = 8 mice). **f** Representative images of eWAT depots. **g** Body composition (n = 8 mice). **h** Energy intake (n = 8 mice). **i, j** Twenty-four hours oxygen consumption (VO_2) (n = 8 mice). **k, l** Respiratory exchange ratio (RER: VCO_2/VO_2) (n = 8 mice). **m** Rectal temperature (n = 8 mice). **n** Representative images of BAT depots. **o** H&E staining of BAT (n = 5 mice; scale bars: 50 μm). **p** UCP1 immunostaining of BAT (n = 5 mice; Scale bars: 100 μm). **q** H&E staining of eWAT (n = 5 mice; scale bars: 50 μm). **r** H&E staining of the liver (n = 5 mice; scale bars: 100 μm). **s** Oil Red O staining of the liver (n = 5 mice; scale bars: 50 μm). **t, u** Blood glucose levels and the AUC of the GTT (**t**) and ITT (**u**) curves (n = 8 mice). 3V, third cerebral ventricle; ARC, arcuate nucleus; GTT, glucose tolerance test; ITT, insulin tolerance test; AUC, area under the curve. The data are expressed as the mean ± SEM. Two-tailed Student's t tests were used in **(c)**; and two-way ANOVA with Bonferroni post-hoc tests were used in **(e, g, h, j l, t, u)**. one-way ANOVA followed by Tukey's test **(m)**. Source data are provided as a Source Data file.

Supplementary Fig. 12

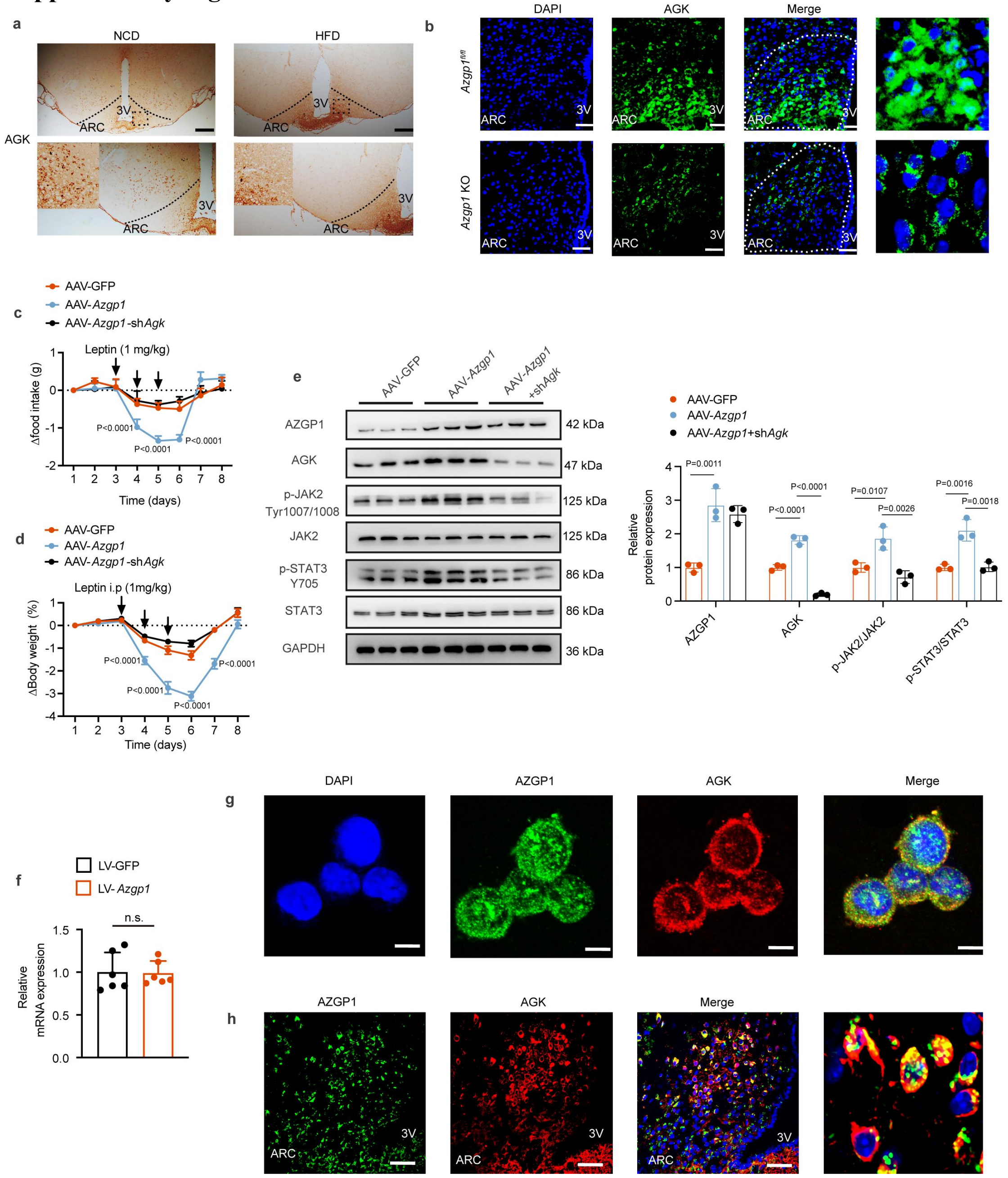

Supplementary Fig. 12 AGK expression is downregulated by HFD in the hypothalamus and is associated with AZGP1. a IHC staining of AGK expression in the hypothalamic ARC of NCD- or HFD-fed WT mice (n = 3 mice; Scale bars: 100μm). **b** IF staining showing AGK expression in the hypothalamic ARC of HFD-fed *Azgp1*$^{fl/fl}$ and POMC-*Azgp1* KO mice (n = 3 mice; Scale bars: 100μm). **c-e** Eight-week-old male WT mice received MBH injection of AAV9-*Azgp1*/ GFP or AVV9-*Azgp1*+AVV9-sh*Agk* and were fed a HFD for 4 weeks. For the

leptin sensitivity experiment, mice were intraperitoneally injected with leptin**.** **c** Changes in food intake (n = 10 mice). **d** Change in body weight (n = 10 mice). **e** Western blots showing AZGP1, AGK, t-JAK2/p-JAK2 (Tyr1007/1008), and t-STAT3/p-STAT3 (Y705) expression in the hypothalamus (n = 3 mice). **f** GT1-7 cells were transfected with or without LV-*Azgp1* as indicated in the Methods. AGK mRNA expression was measured by RT-PCR (n = 6 independent cell experiments). **g** IF staining showing the co-location of AZGP1 and AGK in N2A cells (n = 3 independent cell experiments; Scale bars: 100μm). **h** IF staining showing the colocation of AZGP1 and AGK in the MBH of WT mice (n = 3 independent cell experiments; Scale bars: 100μm). 3V, third cerebral ventricle; ARC, arcuate nucleus; Data were expressed as the mean ± SEM. Two-way ANOVA with Bonferroni post-hoc tests were used in **(c, d)**; one-way ANOVA followed by Tukey's test **(e)**; Two-tailed Student's t tests were used in **(f)** . Source data are provided as a Source Data file. (n.s. not significant.).

Supplemental table

Table S1 Anthropometric characteristics and metabolic indicators in the study population.

Variable	Lean	Overweight	Obesity
N (male/female)	135 (70/65)	38 (20/18)	26 (13/13)
Age (years)	25.5 ± 2.9	26.7 ± 4.0	26.54 ± 5.35
BMI (kg/m^2)	20.0 ± 1.9	25.8 ± 1.0 (p<0.0001)	30.9 ± 2.6 (p<0.0001)
WHR	0.80 ± 0.05	0.85 ± 0.05 (p<0.0001)	0.90 ± 0.05 (p<0.0001)
FAT (%)	26.3 ± 4.9	37.6 ± 3.8 (p<0.0001)	46.3 ± 5.2 (p<0.0001)
TG (mmol/L)	0.97 ± 0.45	1.74 ± 1.06 (p<0.0001)	1.65 ± 0.91 (p<0.0001)
TC (mmol/L)	4.02 ± 1.04	4.47 ± 1.01	4.42 ± 0.98
HDL-C (mmol/L)	1.29 ± 0.59	1.29 ± 0.37	1.29 ± 0.65
LDL-C (mmol/L)	2.24 ± 0.83	2.66 ± 0.91 (p=0.0104)	2.66 ± 0.47 (p=0.0332)
FFA (mmol/L)	0.56 ± 0.26	0.61 ± 0.19	0.61 ± 0.20
FBG (mmol/L)	4.58 ± 0.51	4.90 ± 0.57 (p=0.0035)	4.95 ± 0.70 (p=0.0038)
0.5h-BG (mmol/L)	7.65 ± 1.60	8.54 ± 1.88 (p=0.0070)	9.00 ± 1.47 (p=0.0003)
1h-BG (mmol/L)	6.68 ± 1.98	8.21 ± 2.47 (p=0.0003)	9.90 ± 2.41 (p<0.0001)
2h-BG (mmol/L)	5.79 ± 1.45	7.22 ± 2.30 (p<0.0001)	9.32 ± 2.45 (p<0.0001)
FIns (mU/L)	8.73 ± 4.52	16.9 ± 11.6 (p<0.0001)	27.2 ± 20.9 (p<0.0001)
0.5h-Ins (mU/L)	90.0 ± 63.2	130.3 ± 90.3 (p=0.0025)	124.8 ± 41.4 (p=0.0319)
1h-Ins (mU/L)	76.3 ± 55.1	136.8 ± 84.4 (p<0.0001)	161.7 ± 79.0 (p<0.0001)
2h-Ins (mU/L)	59.6 ± 46.8	130.3 ± 94.2 (p<0.0001)	182.7 ± 91.2 (p<0.0001)
HbA1c (%)	5.23 ± 0.28	5.30 ± 0.33	5.45 ± 0.40 (p=0.0020)

BMI, body mass index; FFA, free fatty acid; FBG, fasting blood glucose; 2h-BG, 2h-blood glucose after oral glucose tolerance test; FIns, Fasting insulin levels; 2h-Ins, 2h-insulin after oral glucose tolerance test; HDL-C, high-density lipoprotein cholesterol; LDL-C, low-density lipoprotein cholesterol; TC, total cholesterol; TG, triglyceride; WHR, waist-hip ratio. Values are given as means ± SD. The statistical signifificance of differences were assessed by one-way ANOVA, followed by Dunnett multiple comparison test.

Table S2 The antibodies used for IF and IHC

Antibody	Source	Dilution
Anti-POMC, rabbit monoclonal	#23499, Cell Signaling Technology	1:500
Anti-AgRP, rabbit monoclonal	ab254558, Abcam	1:500
Anti-AZGP1, mouse monoclonal	sc-271957, Santa Cruz Biotechnology	1:200
Anti-cFos, mouse monoclonal	ab208942, Abcam	1:1000
Anti-AGK, rabbit polyclonal	ab137616, Abcam	1:500
Anti-NeuN, rabbit polyclonal	26975-1-AP, Proteintech	1:500
Anti-GFAP, rabbit polyclonal	16825-1-AP, Proteintech	1:500
Anti-STAT3, rabbit monoclonal	# 12640, Cell Signaling Technology	1:500
Anti-p-STAT3, rabbit monoclonal	# 9145, Cell Signaling Technology	1:500
Goat-anti-mouse IgG, polyclonal	A32723 or A32727, Invitrogen	1:1000
Goat-anti-rabbit IgG, polyclonal	A32732 or A-11034, Invitrogen	1:1000

Table S3 The antibodies used for Western blots

Antibody	Source	Dilution
Anti-AZGP1, rabbit polyclonal	orb354031, Biorbyt Ltd	1:1000
Anti-AGK, rabbit polyclonal	ab137616, Abcam	1:1000
Anti-t-STAT3, mouse monoclonal	#9139s, Cell Signaling Technology	1:1000
Anti-p-STAT3, rabbit monoclonal	#9145s, Cell Signaling Technology	1:1000
Anti-t-JAK2, rabbit monoclonal	#3230s, Cell Signaling Technology	1:1000
Anti-p-JAK2, rabbit polyclonal	#3771s, Cell Signaling Technology	1:1000
Anti-t-FOXO1, rabbit monoclonal	#2880s, Cell Signaling Technology	1:1000
Anti-p-FOXO1, rabbit monoclonal	# 84192s, Cell Signaling Technology	1:1000
Anti-T-mTOR, rabbit monoclonal	#2983s, Cell Signaling Technology	1:1000
Anti-p-mTOR, rabbit monoclonal	#5536s, Cell Signaling Technology	1:1000
Anti-HSL, rabbit monoclonal	#18381, Cell Signaling Technology	1:1000
Anti-p-HSL, mouse polyclonal	#4137, Cell Signaling Technology	1:1000
Anti-p-PKA, rabbit monoclonal	#9624, Cell Signaling Technology	1:1000
Anti-Ub, Rabbit polyclonal	#10201-2-AP, Proteintech	1:1000
Anti-Flag, Rabbit polyclonal	#20543-1-AP, Proteintech	1:2000
Anti-Myc, Rabbit polyclonal	#16286-1-AP, Proteintech	1:2000
Anti-HA, Rabbit polyclonal	#51064-2-AP, Proteintech	1:2000
Anti-GAPDH, Rabbit polyclonal	#5174, Cell Signaling Technology	1:1000
Goat-anti-Rabbit IgG, polyclonal	#SA00001-2, Proteintech	1:8000
Goat-anti-Mouse IgG, polyclonal	#SA00001-1, Proteintech	1:8000

Table S4 The primers used for RT-PCR

Gene Name	Sequence
Pomc	R: CATTAGGCTTGGAGCAGGTC
	F: TCTTGATGATGGCGTTCTTG
Agrp	R: GGCCTCAAGAAGACAACTGC
	F: GCAAAAGGCATTGAAGAAGC
Npy	R: AAGAGCCTGGTCAAGTTCTG
	F: TAGGAGTAGTGCCCAAATGC
Agk	R: GCCGTCTCCTCCAGCAACAATG
	F: AAGAAGAGCAGCCTGTCAAGAAGC
β-actin	R: GGCATAAACGCAGAGCATTCCTG
	F: CAGTGTCCATCCTCTGAGTAGC
Fas	F: CTCATCCACTCAGGTTCAG
	R: AGGTATGCTCGCTTCTCT
Scd-1	F: TGATGTTCCAGAGGAGGTA
	R: CCAGAGTGTATCGCAAGAA
Acc	F: AGCAGTTACACCACATACAT
	R: GTCATCACCATCTTCATTACC
Srebp1c	F: GCTTCTCTTCTGCTTCTCT
	R: GCTGTAGGATGGTGAGTG
Ppar-γ	F: CCGAAGAACCATCCGATT
	R: CGCAGATCAGCAGACTC
Cd36	F: GGTCCTTACACATACAGAGT
	R: CTACAGCCAGATTCAGAACT
Fabp	F: TTCTCAGCCAGCCAGTT
	R: CATCTCCTCGTAAGCCATT
Ppar-α	F: TGCCTTAGAACTGGATGAC
	R: ATCTGGATGGTTGCTCTG
Cpt1a	F: AGCCAGACGAAGAACATC
	R: CCTTGACCATAGCCATCC
Ucp1	F: GAAACACCTGCCTCTCTCGG
	R: GCATTCTGACCTTCACGACCT
Adrβ3	F: TAGCCATCAAACCTGTTGAGC
	R: GGCCCTCTCTAGTTCCCAG
Th	F: CTCTCCTCGAATACCACAGCC
	R: CCAAGGTTCATTGGACGGC
