# Supplementary material for: AZGP1 in POMC neurons modulates energy homeostasis and metabolism through leptin-mediated STAT3 phosphorylation
Source: Nat Commun. 2024 Apr 20;15:3377. doi: 10.1038/s41467-024-47684-9 (PMC11032411; doi:10.1038/s41467-024-47684-9)
Supplement: Supplementary file 3 — Reporting Summary [file 41467_2024_47684_MOESM3_ESM.pdf]

nature portfolio

Corresponding author(s): Gangyi Yang, Ling Li and Mengliu Yang

Last updated by author(s): Mar 25, 2024

# Reporting Summary

Nature Portfolio wishes to improve the reproducibility of the work that we publish. This form provides structure for consistency and transparency in reporting. For further information on Nature Portfolio policies, see our Editorial Policies and the Editorial Policy Checklist.

## Statistics

For all statistical analyses, confirm that the following items are present in the figure legend, table legend, main text, or Methods section.

| n/a | Confirmed | |
|---|---|---|
| [ ] | [x] | The exact sample size ($n$) for each experimental group/condition, given as a discrete number and unit of measurement |
| [ ] | [x] | A statement on whether measurements were taken from distinct samples or whether the same sample was measured repeatedly |
| [ ] | [x] | The statistical test(s) used AND whether they are one- or two-sided<br>*Only common tests should be described solely by name; describe more complex techniques in the Methods section.* |
| [ ] | [x] | A description of all covariates tested |
| [ ] | [x] | A description of any assumptions or corrections, such as tests of normality and adjustment for multiple comparisons |
| [ ] | [x] | A full description of the statistical parameters including central tendency (e.g. means) or other basic estimates (e.g. regression coefficient) AND variation (e.g. standard deviation) or associated estimates of uncertainty (e.g. confidence intervals) |
| [x] | [ ] | For null hypothesis testing, the test statistic (e.g. $F$, $t$, $r$) with confidence intervals, effect sizes, degrees of freedom and $P$ value noted<br>*Give P values as exact values whenever suitable.* |
| [x] | [ ] | For Bayesian analysis, information on the choice of priors and Markov chain Monte Carlo settings |
| [x] | [ ] | For hierarchical and complex designs, identification of the appropriate level for tests and full reporting of outcomes |
| [ ] | [x] | Estimates of effect sizes (e.g. Cohen's $d$, Pearson's $r$), indicating how they were calculated |

*Our web collection on statistics for biologists contains articles on many of the points above.*

## Software and code

Policy information about availability of computer code

| | |
|---|---|
| Data collection | Animal monitoring system (clams; Oxymax, Columbus, USA)<br>Rectal temperature (Physitemp Instruments)<br>Fluorescence microscope (BX53F , Olympus)<br>Nikon confocal microscope (Ti2E, Japan)<br>RT–PCR was performed with the CFX ConnectTM Optics Module (Bio-Rad)<br>Vibratome (Leica VT1200S)<br>IR-DIC optical system (Eclipse FN-1, Nikon) |
| Data analysis | SPSS software (version 25.0)<br>Prism software version 9.0 (GraphPad)<br>Electrophysiological signals were recorded and analyzed by a MultiClamp 700B amplifier (Axon Instruments) and Clampfit 10.3 software (Molecular Devices) |

For manuscripts utilizing custom algorithms or software that are central to the research but not yet described in published literature, software must be made available to editors and reviewers. We strongly encourage code deposition in a community repository (e.g. GitHub). See the Nature Portfolio guidelines for submitting code & software for further information.

# Data

Policy information about availability of data

All manuscripts must include a data availability statement. This statement should provide the following information, where applicable:

- Accession codes, unique identifiers, or web links for publicly available datasets
- A description of any restrictions on data availability
- For clinical datasets or third party data, please ensure that the statement adheres to our policy

All data generated during this study are provided with this paper in the source data files. Source data are provided with this paper. Mouse brain atlas are available: https://searchworks.stanford.edu/view/9860513.

# Research involving human participants, their data, or biological material

Policy information about studies with human participants or human data. See also policy information about sex, gender (identity/presentation), and sexual orientation and race, ethnicity and racism.

**Reporting on sex and gender**

A total of 199 subjects were enrolled in this study including 99 males and 100 females.

**Reporting on race, ethnicity, or other socially relevant groupings**

All subjects from the Second Affiliated Hospital of Chongqing Medical University. there is no potential bias for recruiting patient cohort.

**Population characteristics**

The clinical information of participants included in this study was shown in Supplementary Table 1. The information was also shown below.

| Variable | Lean | Overweight | Obesity |
|---|---|---|---|
| Age (years) | 25.5 ± 2.9 | 26.7 ± 4.0 | 26.54 ± 5.35 |
| BMI (kg/m2) | 20.0 ± 1.9 | 25.8 ± 1.0 | 30.9 ± 2.6 |
| WHR | 0.80 ± 0.05 | 0.85 ± 0.05 | 0.90 ± 0.05 |
| FAT (%) | 26.3 ± 4.9 | 37.6 ± 3.8 | 46.3 ± 5.2 |
| TG (mmol/L) | 0.97 ± 0.45 | 1.74 ± 1.06 | 1.65 ± 0.91 |
| TC (mmol/L) | 4.02 ± 1.04 | 4.47 ± 1.01 | 4.42 ± 0.98 |
| HDL-C (mmol/L) | 1.29 ± 0.59 | 1.29 ± 0.37 | 1.29 ± 0.65 |
| LDL-C (mmol/L) | 2.24 ± 0.83 | 2.66 ± 0.91 | 2.66 ± 0.47 |
| FFA (μmol/L) | 0.56 ± 0.26 | 0.61 ± 0.19 | 0.61 ± 0.20 |
| FBG (mmol/L) | 4.58 ± 0.51 | 4.90 ± 0.57 | 4.95 ± 0.70 |
| 0.5h-BG (mmol/L) | 7.65 ± 1.60 | 8.54 ± 1.88 | 9.00 ± 1.47 |

**Recruitment**

Individuals were recruited from the Second Affiliated Hospital of Chongqing Medical University from 2018 to 2021. None of the individuals took any medication or underwent a lifestyle intervention. Individuals with any other disease were excluded from the study.

**Ethics oversight**

The work was approved by the Research Ethics Committee of Chongqing Medical University.

Note that full information on the approval of the study protocol must also be provided in the manuscript.

# Field-specific reporting

Please select the one below that is the best fit for your research. If you are not sure, read the appropriate sections before making your selection.

☒ Life sciences ☐ Behavioural & social sciences ☐ Ecological, evolutionary & environmental sciences

For a reference copy of the document with all sections, see nature.com/documents/nr-reporting-summary-flat.pdf

# Life sciences study design

All studies must disclose on these points even when the disclosure is negative.

**Sample size**

Sample size is indicated in the figure legend in each experiment. Animal sample sizes (PMID: 30143607; PMID: 22201683; PMID: 31222048; PMID: 28924165) and Cell numbers (PMID :26923837; PMID: 27207522 )of relative experiments in this study were determined based on previous studies in this field.

**Data exclusions**

No data were excluded

**Replication**

All experiments in vitro and in cell based models were performed at least three time independently. In vivo mouse models used at least 5 mice per genotype. All experimental repetitions were successfully performed.

**Randomization**

All samples were randomly allocation.

Blinding

The experiments were not blinded. However, we followed standard laboratory procedures of randomization. Each experiment was associated with the proper controls, and compared samples were collected and analyzed under the same condition.

# Reporting for specific materials, systems and methods

We require information from authors about some types of materials, experimental systems and methods used in many studies. Here, indicate whether each material, system or method listed is relevant to your study. If you are not sure if a list item applies to your research, read the appropriate section before selecting a response.

## Materials & experimental systems

| n/a | Involved in the study |
|---|---|
| ☐ | ☒ Antibodies |
| ☐ | ☒ Eukaryotic cell lines |
| ☒ | ☐ Palaeontology and archaeology |
| ☐ | ☒ Animals and other organisms |
| ☒ | ☐ Clinical data |
| ☒ | ☐ Dual use research of concern |
| ☒ | ☐ Plants |

## Methods

| n/a | Involved in the study |
|---|---|
| ☒ | ☐ ChIP-seq |
| ☒ | ☐ Flow cytometry |
| ☒ | ☐ MRI-based neuroimaging |

## Antibodies

Antibodies used

The antibodies used for IF and IHC
Anti-POMC, rabbit monoclonal #23499, Cell Signaling Technology 1:500
Anti-AgRP, rabbit monoclonal ab254558, Abcam 1:500
Anti-AZGP1, mouse monoclonal sc-271957, Santa Cruz Biotechnology 1:200
Anti-cFos, mouse monoclonal ab208942, Abcam 1:1000
Anti-AGK, rabbit polyclonal ab137616, Abcam 1:500
Anti-NeuN, rabbit polyclonal 26975-1-AP, Proteintech 1:500
Anti-GFAP, rabbit polyclonal 16825-1-AP, Proteintech 1:500
Anti-STAT3, rabbit monoclonal # 12640, Cell Signaling Technology 1:500
Anti-p-STAT3, rabbit monoclonal # 9145, Cell Signaling Technology 1:500
Goat-anti-mouse IgG, polyclonal A32723 or A32727, Invitrogen 1:1000
Goat-anti-rabbit IgG, polyclonal A32732 or A-11034, Invitrogen 1:1000
The antibodies used for Western blots
Anti-AZGP1, rabbit polyclonal orb354031, Biorbyt Ltd 1:1000
Anti-AGK, rabbit polyclonal ab137616, Abcam 1:1000
Anti-t-STAT3, mouse monoclonal #9139s, Cell Signaling Technology 1:1000
Anti-p-STAT3, rabbit monoclonal #9145s, Cell Signaling Technology 1:1000
Anti-t-JAK2, rabbit monoclonal #3230s, Cell Signaling Technology 1:1000
Anti-p-JAK2, rabbit polyclonal #3771s, Cell Signaling Technology 1:1000
Anti-t-FOXO1, rabbit monoclonal #2880s, Cell Signaling Technology 1:1000
Anti-p-FOXO1, rabbit monoclonal # 84192s, Cell Signaling Technology 1:1000
Anti-T-mTOR, rabbit monoclonal #2983s, Cell Signaling Technology 1:1000
Anti-p-mTOR, rabbit monoclonal #5536s, Cell Signaling Technology 1:1000
Anti-HSL, rabbit monoclonal #18381, Cell Signaling Technology 1:1000
Anti-p-HSL, mouse polyclonal #4137, Cell Signaling Technology 1:1000
Anti-p-PKA, rabbit monoclonal #9624, Cell Signaling Technology 1:1000
Anti-Ub, Rabbit polyclonal #10201-2-AP, Proteintech 1:1000
Anti-Flag, Rabbit polyclonal #20543-1-AP, Proteintech 1:2000
Anti-Myc, Rabbit polyclonal #16286-1-AP, Proteintech 1:2000
Anti-HA, Rabbit polyclonal #51064-2-AP, Proteintech 1:2000
Anti-GAPDH, Rabbit polyclonal #5174, Cell Signaling Technology 1:1000
Goat-anti-Rabbit IgG, polyclonal #SA00001-2, Proteintech 1:8000
Goat-anti-Mouse IgG, polyclonal #SA00001-1, Proteintech 1:8000

Validation

Anti-POMC (https://www.cellsignal.cn/products/primary-antibodies/pomc-d3r1u-rabbit-mab/23499)
Anti-AgRP (https://www.abcam.cn/products/primary-antibodies/agrp-antibody-epr18155-110-ab254558.html)
Anti-AZGP1 (https://www.scbt.com/p/zag-antibody-f-6?requestFrom=search)
Anti-cFos (https://www.abcam.cn/products/primary-antibodies/c-fos-antibody-2h2-ab208942.html)
Anti-AGK (https://www.abcam.cn/products?keywords=Anti-AGK)
Anti-NeuN (https://www.ptgcn.com/products/NeuN-Antibody-26975-1-AP.htm)
Anti-GFAP (https://www.ptgcn.com/products/GFAP-Antibody-16825-1-AP.htm)
Anti-STAT3 (https://www.cellsignal.cn/products/primary-antibodies/stat3-d3z2g-rabbit-mab/12640)

Anti-p-STAT3 (https://www.cellsignal.cn/products/primary-antibodies/phospho-stat3-tyr705-d3a7-xp-rabbit-mab/9145)
Goat-anti-mouse IgG (https://www.thermofisher.cn/cn/zh/antibody/product/Goat-anti-Mouse-IgG-H-L-Highly-Cross-Adsorbed-Secondary-Antibody-Polyclonal/A32723)/ ( https://www.thermofisher.cn/cn/zh/antibody/product/Goat-anti-Mouse-IgG-H-L-Highly-Cross-Adsorbed-Secondary-Antibody-Polyclonal/A32727)
Goat-anti-rabbitIgG(https://www.thermofisher.cn/cn/zh/antibody/product/Goat-anti-Rabbit-IgG-H-L-Highly-Cross-Adsorbed-Secondary-Antibody-Polyclonal/A32732)/(https://www.thermofisher.cn/cn/zh/antibody/product/Goat-anti-Rabbit-IgG-H-L-Highly-Cross-Adsorbed-Secondary-Antibody-Polyclonal/A-11034)
Anti-AZGP1 (https://www.biorbyt.com/azgp1-antibody-orb354031.html)
Anti-AGK (https://www.abcam.cn/products?keywords=Anti-AGK)
Anti-t-STAT3 (https://www.cellsignal.cn/products/primary-antibodies/stat3-124h6-mouse-mab/9139)
Anti-p-STAT3(https://www.cellsignal.cn/products/primary-antibodies/phospho-stat3-tyr705-d3a7-xp-rabbit-mab/9145)
Anti-t-JAK2 (https://www.cellsignal.cn/products/primary-antibodies/jak2-d2e12-xp-rabbit-mab/3230)
Anti-p-JAK2 (https://www.cellsignal.cn/products/primary-antibodies/phospho-jak2-tyr1007-1008-antibody/3771)
Anti-t-FOXO1 (https://www.cellsignal.cn/products/primary-antibodies/foxo1-c29h4-rabbit-mab/2880)
Anti-p-FOXO1(https://www.cellsignal.cn/products/primary-antibodies/phospho-foxo1-ser256-e1f7t-rabbit-mab/84192)
Anti-T-mTOR (https://www.cellsignal.cn/products/primary-antibodies/mtor-7c10-rabbit-mab/2983)
Anti-p-mTOR(https://www.cellsignal.cn/products/primary-antibodies/phospho-mtor-ser2448-d9c2-xp-rabbit-mab/5536)
Anti-HSL (https://www.cellsignal.cn/products/primary-antibodies/hsl-d6w5s-xp-rabbit-mab/18381)
Anti-p-HSL(https://www.cellsignal.cn/products/primary-antibodies/phospho-hsl-ser565-antibody/4137)
(https://www.cellsignal.cn/products/primary-antibodies/phospho-pka-substrate-rrxs-t-100g7e-rabbit-mab/9624)
Anti-Ub (https://www.ptgcn.com/products/ubiquitin-Antibody-10201-2-AP.htm)
Anti-Flag (https://www.ptgcn.com/products/Flag-Tag-Antibody-20543-1-AP.htm)
Anti-Myc (https://www.ptgcn.com/products/MYC-tag-Antibody-16286-1-AP.htm)
Anti-HA (https://www.ptgcn.com/products/HA-tag-Antibody-51064-2-AP.htm)
Anti-GAPDH (https://www.cellsignal.cn/products/primary-antibodies/gapdh-d16h11-xp-rabbit-mab/5174)
Goat-anti-RabbitIgG (https://www.ptgcn.com/products/HRP-conjugated-Affinipure-Goat-Anti-Rabbit-IgG-H-L-secondary-antibody.htm)
Goat-anti-MouseIgG(https://www.ptgcn.com/products/HRP-conjugated-Affinipure-Goat-Anti-Mouse-IgG-H-L-secondary-antibody.htm)

# Eukaryotic cell lines

Policy information about cell lines and Sex and Gender in Research

| | |
|---|---|
| Cell line source(s) | N2A cells (SCSP-5035) and HEK293T (GNHu44) were purchased from National Collection of Authenticated Cell Cultures. GT1-7 cells (9Q01F4) was purchased from MERCK. |
| Authentication | None of the cell lines we used was authenticated by ourself. |
| Mycoplasma contamination | All cell lines used in this study were regularly tested for mycoplasma contamination and were determined to be negative. |
| Commonly misidentified lines (See ICLAC register) | No misidentified lines were used. |

# Animals and other research organisms

Policy information about studies involving animals; ARRIVE guidelines recommended for reporting animal research, and Sex and Gender in Research

| | |
|---|---|
| Laboratory animals | Eight-week-old male C57 BL/6J mice were acquired from GemPharmatech Co., Ltd. (Jiangsu, China). POMC-Cre mice, tamoxifen-inducible POMC-Cre (POMC- CreER) mice and Rosa26-tdTomato (tdTomato) reporter mice were provided by Prof. Guo (Chinese Academy of Sciences, Shanghai, China). Stat3flox/flox mice were provided by Dr. Liu (Huazhong University of Science and Technology, Wuhan, China). Azgp1fl/fl (S-CKO-01376), AgRP-Cre (C001249), ObRb-Cre (C001036) and ob/ob mice were obtained from Cyagen Biotechnology Co., Ltd. (Jiangsu, China). All mice were on a C57BL/6 background. All mice were housed at a controlled room temperature of 25 °C and humidity (40-60%) with a 12-h light/dark cycle, with water and food provided. POMC-CreER mice were crossed with tdTomato reporter mice to generate mice with tdTomato-labeled POMC neurons (POMC-CreER-tdTomato mice). Azgp1fl/fl mice or Stat3fl/fl mice were crossed with POMC-CreER-tdTomato mice to produce POMC-Azgp1 KO and POMC-Stat3 KO mice expressing tdTomato, respectively. To establish diet-induced obesity or IR animal models, 8-week-old male mice were fed a NCD (containing 15.75% of calories from fat, AIN-93G, Xietong Pharmaceutical Bio-engineering Co., Ltd;) or HFD (60% fat, D12492, Research Diets, New Brunswick, NJ) for 4 or 12 weeks. To induce adult-onset gene deletion in POMC neurons, 6-week-old male POMC-Azgp1 KO or POMC-Stat3 KO mice were injected with tamoxifen (0.15 g/kg) intraperitoneally for 14 days. |
| Wild animals | No wild animals were used in this study. |
| Reporting on sex | Adult male and female mice were used in our experiments. |
| Field-collected samples | No field-collected samples were used in this study. |
| Ethics oversight | All procedures were approved by the Animal Experimentation Ethics Committee, Chongqing Medical University. |

Note that full information on the approval of the study protocol must also be provided in the manuscript.

## Plants

Seed stocks

*Report on the source of all seed stocks or other plant material used. If applicable, state the seed stock centre and catalogue number. If plant specimens were collected from the field, describe the collection location, date and sampling procedures.*

Novel plant genotypes

*Describe the methods by which all novel plant genotypes were produced. This includes those generated by transgenic approaches, gene editing, chemical/radiation-based mutagenesis and hybridization. For transgenic lines, describe the transformation method, the number of independent lines analyzed and the generation upon which experiments were performed. For gene-edited lines, describe the editor used, the endogenous sequence targeted for editing, the targeting guide RNA sequence (if applicable) and how the editor was applied.*

Authentication

*Describe any authentication procedures for each seed stock used or novel genotype generated. Describe any experiments used to assess the effect of a mutation and, where applicable, how potential secondary effects (e.g. second site T-DNA insertions, mosiacism, off-target gene editing) were examined.*
